# Transcranial bright light treatment via the ear canals in seasonal affective disorder: a randomized, double-blind dose-response study

**DOI:** 10.1186/s12888-014-0288-6

**Published:** 2014-10-21

**Authors:** Heidi Jurvelin, Timo Takala, Juuso Nissilä, Markku Timonen, Melanie Rüger, Jari Jokelainen, Pirkko Räsänen

**Affiliations:** Department of Psychiatry, University of Oulu, Institute of Clinical Medicine, Box 5000, 90014 Oulu, Finland; University of Oulu, Institute of Health Sciences, Box 5000, 90014 Oulu, Finland; Valkee Oy, Elektroniikkatie 4, 90590 Oulu, Finland; Oulu Deaconess Institute, Box 365, 90101 Oulu, Finland; Oulu Health Center, Box 8, 90015 Oulu, Finland; Unit of General Practice, Oulu University Hospital, 90029 Oulu, Finland; Department of Psychiatry, Oulu University Hospital, Box 26, 90026 Oulu, Finland

**Keywords:** Anxiety, Depression, Cognition, Transcranial bright light, Bright light therapy, BDI, HAMA, SIGH-SAD

## Abstract

**Background:**

Bright light treatment is effective for seasonal affective disorder (SAD), although the mechanisms of action are still unknown. We investigated whether transcranial bright light via the ear canals has an antidepressant effect in the treatment of SAD.

**Methods:**

During the four-week study period, 89 patients (67 females; 22 males, aged 22-65, mean ± SD age: 43.2 ± 10.9 years) suffering from SAD were randomized to receive a 12-min daily dose of photic energy of one of three intensities (1 lumen/0.72 mW/cm^2^; 4 lumens/2.881 mW/cm^2^; 9 lumens/6.482 mW/cm^2^) via the ear canals. The light was produced using light-emitting diodes. The severity of depressive symptoms was assessed with the Hamilton Depression Rating Scale – Seasonal Affective Disorder (SIGH-SAD), the Hamilton Anxiety Rating Scale (HAMA), and the Beck Depression Inventory (BDI). Cognitive performance was measured by the Trail Making Test (TMT). The within-group and between-group changes in these variables throughout the study were analysed with a repeated measures analysis of variance (ANOVA), whereas gender differences at baseline within the light groups were analysed using Student’s t-tests.

**Results:**

Patients in all three groups showed significant decreases in their BDI, HAMA, and SIGH-SAD scores. Response rates, i.e., an at least 50% decrease of symptoms as measured by the BDI, were 74%-79% in the three treatment groups. Corresponding variations for the SIGH-SAD and the HAMA were 35-45% and 47-62%, respectively. No intensity-based dose-response relationships in the improvement of anxiety and depressive symptoms or cognitive performance between treatment groups were observed. Approximately one in four patients experienced mild adverse effects, of which the most common were headache, insomnia, and nausea.

**Conclusions:**

These results suggests that transcranial bright light treatment may have antidepressant and anxiolytic effect in SAD patients, as both self- and psychiatrist-rated depressive and anxiety symptoms decreased in all treatment groups. These improvements are comparable to findings of earlier bright light studies that used conventional devices. The lack of dose response may be due to a saturation effect above a certain light intensity threshold. Further studies on the effects of transcranial bright light with an adequate placebo condition are needed.

**Trial registration:**

NCT01293409, ClinicalTrials.gov

## Background

The seasonal pattern of recurrent episodes of depression is known as seasonal affective disorder (SAD) [1–3]. The precise pathogenesis of SAD is uncertain, despite several explanatory theories such as photoperiod and phase-shifted circadian rhythms, neurotransmitter functions, and/or a genetic basis [4,5]. Given the fact that winter SAD is far more prevalent than summer SAD [2,6], the term SAD usually refers to winter SAD and is used accordingly in this article hereafter. Episodes of SAD are characterized by typical and atypical depressive symptoms, i.e., lowered mood, energy loss, excessive sleep with difficulty waking, cravings for carbohydrates, weight gain, irritability, social withdrawal, daytime fatigue, and loss of concentration [7,8]. Cognitive functioning is also impaired in patients suffering from SAD [9].

The prevalence of SAD varies from 0% to 9.7% in the general population [6]. In Western Europe, e.g., Finland, Germany, England, and the Netherlands, the prevalence of SAD has been reported to be approximately 7.1%, 12.7%, 4.4%, and 3.3%, respectively [10,11]. Climatological, geographical latitude, social, and cultural influences and genetic factors have been reported to have an impact on the prevalence of SAD [6,8,12].

Bright light therapy (BLT) has been found to be effective in the treatment of SAD [13-15]. In a meta-analysis, the effect size for the reduction of depressive symptoms was 0.84 [16]. The antidepressant effect of BLT seems to be potentiated by early morning administration [17]. According to clinical guidelines, the recommended bright light exposure for the treatment of SAD is 10,000 lux for 30 min per day when traditional fluorescent light sources are used [18]. Lower-intensity short-wavelength LED light sources may be comparably effective for the treatment of SAD [19].

The mechanism of action of BLT in the treatment of SAD is under debate [20,21]. According to the prevailing theory, the non-image forming (NIF) effects of light are mediated by a retinal photoreceptor system [22–24]. Melanopsin (OPN4), which is found in the intrinsically photosensitive retinal ganglion cells (ipRGCs), is mainly responsible for this phototransduction [25,26]. However, the classical visual photoreceptor systems have also been shown to play important roles in the transmission of non-visual photic responses in mammals [27]. Light-induced neural signals from the ipRGCs are transmitted to the suprachiasmatic nucleus (SCN) via the retinohypothalamic tract (RHT), [28]. Recently, it has been reported that OPN4 gene variants may dispose some individuals to SAD [29].

However, potentially light sensitive opsins, e.g., melanopsin, encephalopsin (OPN3), and neuropsin (OPN5) have also been found at the mRNA and/or protein level in several areas of the human [30–32] and rodent brain [32–36].

Light seems to be able to penetrate the mammalian brain [37], including the human brain [38]. Our recent findings suggest that transcranial bright light (TBL) treatment via the ear canals modulates the neural networks of the human brain [39], improves cognitive performance in healthy participants [40,41], alleviates symptoms of SAD [42], and acutely affects cardiovascular autonomic regulation [43]. Moreover, TBL stimulation from one side of the skull seems to change the functional connectivity of the human brain as measured by Quantitative Electroencephalography (QEEG) and low resolution electromagnetic tomography (LORETA) [38]. In addition, cortical photostimulation seems to have effects on immunoregulation on patients suffering from rheumatoid arthritis [44].

The effects of transcranially administered light are unlikely to be mediated via the eyes for several reasons. There is light-absorbing melanin in the back of the eye. Conscious visual perception of light has not been reported by any participants during transcranial illumination in an earlier study, and no eyesight-induced changes in the visual cortex were observed by fMRI [39]. Moreover, a recently published study showed that transcranial bright light administered in the evening does not suppress melatonin secretion [45]. The ability of light to suppress melatonin secretion is well-established to be mediated via the RHT [46]. Thus, we hypothesize that some effects of external light are mediated via routes without the function of the eyes.

The aim of this study was to investigate the effects of TBL treatment administered via the ear canals on depressive and anxiety symptoms in patients suffering from SAD in a randomized, double-blind dose-response study design.

## Methods

**Figure 1:**
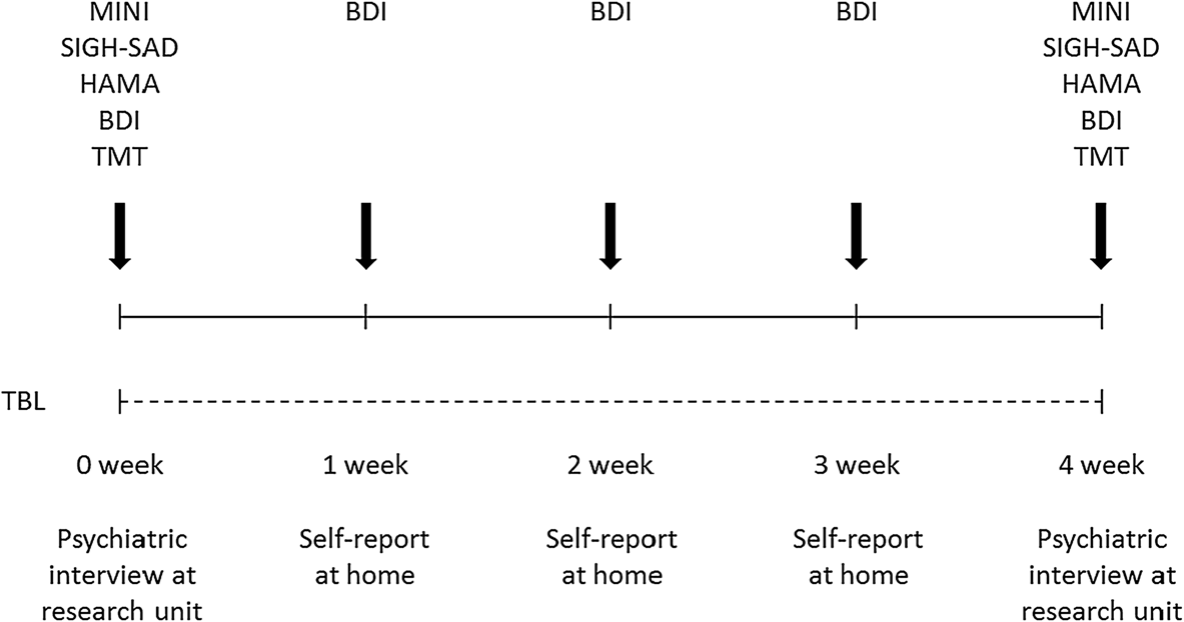
**Randomized study procedures.** A 12-minute bright light dose was administered transcranially via the ear canals daily after awakening. The intensity of the transcranial bright light (TBL) was 1, 4 and 9 lumens in treatment groups 1, 2, and 3, respectively. Depressive symptoms were evaluated using the SIGH-SAD, HAMA, and BDI measurements. Cognitive performance was measured using the Trail-Making Test (TMT) part A and B.

The study was conducted during the darkest period of the year (from the 24th of November to the 4th of March) in northern Finland (latitude 65°N), where the shortest daylight duration was less than 4 hours. Ninety adults suffering from seasonal depressive symptoms were recruited through advertisements in a local newspaper and pre-screened for symptoms of SAD by a phone interview. The experimental study design is presented in Figure 1. Structured diagnostic interviews were conducted at week 0 and 4 by two trained psychiatrists. The patients were instructed to avoid alcohol for 24 h and coffee and other caffeine-rich drinks for 3 h preceding the 0- and 4-week measurements. The patients were also instructed to go to bed no later than 11 PM the night before the 0- and 4-week measurements. All measurements were conducted at approximately the same time of day to minimize the influence of diurnal variation in the outcome measurements. Ambient light was kept constant in the research room during all visits. A diagnosis for recurrent major depression (moderate or severe) according to the Diagnostic and Statistical Manual of Mental Disorders (DSM-IV) [1] was obtained using the Mini International Neuropsychiatric Interview (MINI) [47]. In addition, patients had to fulfil the diagnostic criteria for “seasonal pattern” [1].

Inclusion criteria were as follows: patients had to have a score of at least 20 on the 29-item Structured Interview Guide for the Hamilton Depression Rating Scale – Seasonal Affective Disorder (SIGH-SAD) [48], a score of 10 or more on the 21-item Hamilton Depression Rating Scale (HAMD), and a score of 5 or more on the eight-item atypical symptom score. Our inclusion criteria were analogous with the criteria used for evaluating the response to treatment in patients with SAD in previous studies [13,49]. Patients with lifetime psychotic disorders, bipolar disorders, severe personality disorders, substance abuse or dependence, suicidal ideation during the past month, any psychotropic medications, and other bright-light therapy for the current SAD episode were excluded from this study. Pregnant females were also excluded. Written informed consent was obtained from the patients after they had been given a full description of the study at the first visit. The research protocol was approved by the Ethics Committee of Oulu University Hospital, Finland, in compliance with the Declaration of Helsinki. This study is registered with ClinicalTrials.gov, number NCT01293409.

The bright light treatment was administered transcranially via the ear canals using a bright light device (Valkee Ltd., Oulu, Finland). The blue-enriched bright light was produced using two light-emitting diodes (LEDs), and was transmitted into both ear canals by an optical fibre. The patients were instructed to administer the TBL treatment daily at home shortly after they normally awakened, and always before noon. Based on an earlier study [42], the duration of the treatment was fixed to 12 minutes in the device settings. Patients were carefully instructed to turn on the treatment device only when the ear plugs were inserted in the ear canal. After 12 minutes, the treatment device turned off automatically. Subjects were not allowed to use any other light therapy devices during the study period.

**Figure 2:**
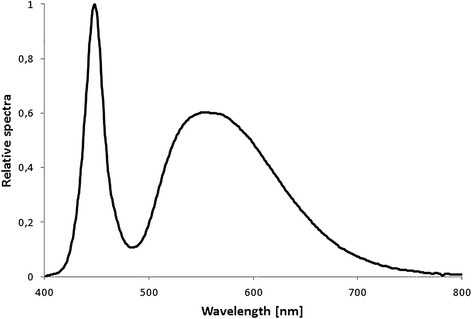
**Spectral power distribution of the bright light produced by the light-emitting diode (LED) of the randomly selected device.**

Patients were randomized into three groups: low dosage (group 1), intermediate dosage (group 2), and high dosage TBL (group 3). The randomization process was planned and implemented in practice by a person from outside of the research group. Both the research team and the patients were blind to the group assignment and receiving active treatment. The luminous intensities of the LEDs in the three groups were 1 lumen, 4 lumens, and 9 lumens, respectively. Lumen is a unit for luminous flux, which is defined as the total amount of visible light emitted from a light source through a solid angle. The corresponding irradiances (μW/cm^2^) and photon densities (photons/cm^2^/s) are presented in Table 1. Figure 2 shows the power spectra of the devices measured directly from a distance of 1 cm.

**Table 1:** **Radiometric and photometric comparisons of the light devices used in the three treatment groups**

**Group**	**Spectral characteristics**	**Luminous flux (lumen)**	**Illuminance (lux)**	**Irradiance (μW/cm** ^**2**^ **)**	**Photon density (photons/cm** ^**2**^ **/s)**
1	λ_max_ ≈ 448 nm	1	2386	720	2.00 × 10^15^
2	λ_max_ ≈ 448 nm	4	9542	2881	7.98 × 10^15^
3	λ_max_ ≈ 448 nm	9	21470	6482	1.80 × 10^16^

Values are measured and calculated from one randomly selected light source. Illuminance and photon density are measured at a distance of 1 cm from the light source. There were minor differences in the spectral compositions between light sources.

The sum scores of the 21-item Beck Depression inventory (BDI) [50], the 29-item SIGH-SAD, and the 14-item Structured Interview Guide for the Hamilton Anxiety Rating Scale (HAMA) [51] were used to evaluate the severity of SAD. The criterion for response was fulfilled when the patient achieved a 50% or more decrease from the baseline BDI, SIGH-SAD, and HAMA scores. The remission rate was defined as a total score ≤ the cut-off and the decrease of symptoms by 50% or more. The cut-off used for the SIGH-SAD and the BDI in this study were 8 and 10, respectively. In addition, information about adverse events related to the bright light treatment was recorded.

Cognitive functioning was measured by the Trail Making Test (TMT) [52] at week 0 and 4. The TMT provides information on visual search, scanning, speed of processing, mental flexibility, and executive functions [53]. The TMT consists of two parts. In part A, the patient is asked to draw lines to connect encircled numbers in a numerical sequence (i.e., 1–2–3, etc.). In part B, the patient is asked to draw lines to connect encircled numbers and letters in an alternating numeric and alphabetic sequence (i.e., 1-A–2-B, etc.) as fast as possible [54]. The score on each part represents the amount of time required to complete the task. The TMT-A test measures attention and concentration while the TMT-B test measures mental flexibility [55].

All data are presented as percentages or as the mean and 95% confidence intervals. Student’s t-tests were used to compare baseline values between genders within the light treatment groups. Categorical variables were compared by either a Chi-Square test or Fisher’s Exact test as appropriate. The within-group and between-group changes in variables during the study were analysed with repeated measures analysis of variance (ANOVA). Results with two-tailed p-values <0.05 were considered statistically significant. Statistical analyses were performed using SAS version 9.2 (SAS Institute Inc, Cary, NC, USA).

## Results

One patient was excluded from the study due to an unexpected trip abroad (N = 89). The mean age ± SD of the remaining patients was 43.0 ± 10.9 years (age range: 22 to 65 years). Subject characteristics were similar across groups in most respects but differed for some variables. Statistically significant differences were found for the variables age (p = 0.0045) and BDI baseline sum score (p = 0.0185) in treatment group 3 and for the SIGH-SAD baseline sum score (p = 0.0414) in treatment group 1 between females and males.

### Depression scores

When compared to baseline (week 0), statistically significant reductions were found for the mean BDI, SIGH-SAD, and HAMA total scores after adjusting for age and gender in each treatment group. Total scores in week 0 and 4 are presented in Table 2. The percentage improvement measured by the BDI varied from 63% to 67% in the three treatment groups. Corresponding variations for the SIGH-SAD and the HAMA were 44%-47% and 47%-50%, respectively (Table 3). There were no significant differences in improvements between groups.

**Table 2:** **Depression and anxiety scale scores as measured by the SIGH-SAD, HAMA, and BDI**

**Measure**	**Group 1 (n = 28)**		**Group 2 (n = 31)**		**Group 3 (n = 30)**	
	**Mean**	**95% CI**	**Mean**	**95% CI**	**Mean**	**95% CI**
SIGH-SAD						
Week 0	36.6	34.1-39.0	36.1	33.7-38.5	35.7	33.5-37.8
Week 4	19.0	14.4-23.6	19.1	15.5-22.7	19.8	15.6-23.9
HAMD						
Week 0	21.8	20.2-23.5	21.5	19.5-23.5	21.1	19.6-22.6
Week 4	11.3	8.5-14.1	11.1	9.0-13.1	11.5	8.9-14.0
Atypical score						
Week 0	14.8	13.1-16.4	14.5	13.2-15.9	14.6	13.2-16.0
Week 4	7.7	5.3-10.1	8.0	6.2-9.9	8.3	6.3-10.3
HAMA						
Week 0	23.6	21.3-26.0	22.6	20.2-25.1	22.1	19.9-24.2
Week 4	11.6	8.2-14.9	11.2	8.7-13.7	12.0	9.1-14.8
BDI						
Week 0	20.6	17.5-23.7	18.9	15.8-22.1	19.3	15.8-22.9
Week 4	6.9	3.6-10.1	5.5	3.2-7.9	7.4	4.4-10.4

**Table 3:** **Improvement of symptoms as measured by the SIGH-SAD, HAMA, and BDI**

**Improvement**	**Group 1 (n = 28)**			**Group 2 (n = 31)**			**Group 3 (n = 30)**		
	**%**	**95% CI**	**p-value**	**%**	**95% CI**	**p-value**	**%**	**95% CI**	**p-value**
SIGH-SAD	47.4	34.9-60.0	0.0001	45.9	36.0-55.7	0.0001	43.7	32.7-54.8	0.0001
HAMA	49.9	34.7-65.2	0.0137	49.5	38.5-60.5	0.0056	46.5	35.9-57.2	0.0001
BDI	67.3	53.0-81.6	0.0158	67.4	55.5-79.4	0.1282	63.2	49.9-76.6	0.0013

**Figure 3:**
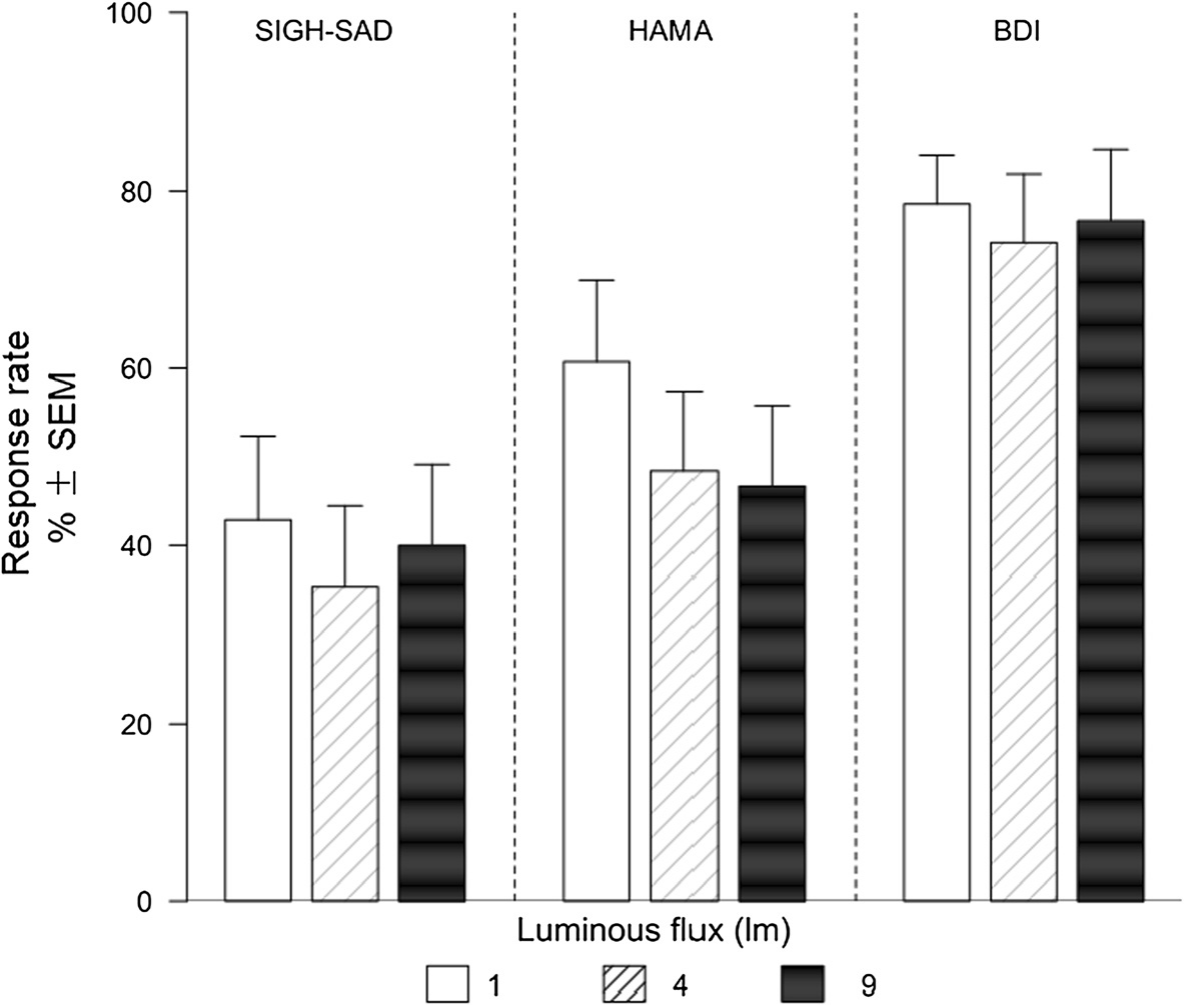
**Response rates measured by SIGH-SAD, HAMA and BDI.** Response rates are defined as the percentage of patients who met the response criteria, i.e., a >50% decrease of symptom scores. Values are reported as% ± sem.

The percentages of the patients in each group who achieved 50% or greater improvement in SAD symptoms are shown in Figure 3. The response rates measured by the BDI varied from 74% to 79% in the three treatment groups. Corresponding variations for the SIGH-SAD and the HAMA were 35-45% and 47-62%, respectively. Response rates did not differ significantly across treatment groups.

Remission rates, as measured by self-rated BDI, ranged from 71% to 79% in the three treatment groups (Table 4). The corresponding variation for the SIGH-SAD ranged from 13% to 29%. Remission rates did not differ significantly across treatment groups.

**Table 4:** **Remission rates as measured by the SIGH-SAD and BDI**

**Remission rate**	**Group 1 (n = 28)**		**Group 2 (n = 31)**		**Group 3 (n = 30)**	
	**Proportion (%)**	**95% CI**	**Proportion (%)**	**95% CI**	**Proportion (%)**	**95% CI**
^1^SIGH-SAD	28.6	10.7-46.6	16.1	2.4-29.8	13.3	0.4-26.2
^2^BDI	78.6	63.1-94.1	71.0	54.7-87.3	76.7	61.2-92.1

^1^cut-off =8.

^2^cut-off =10.

**Figure 4:**
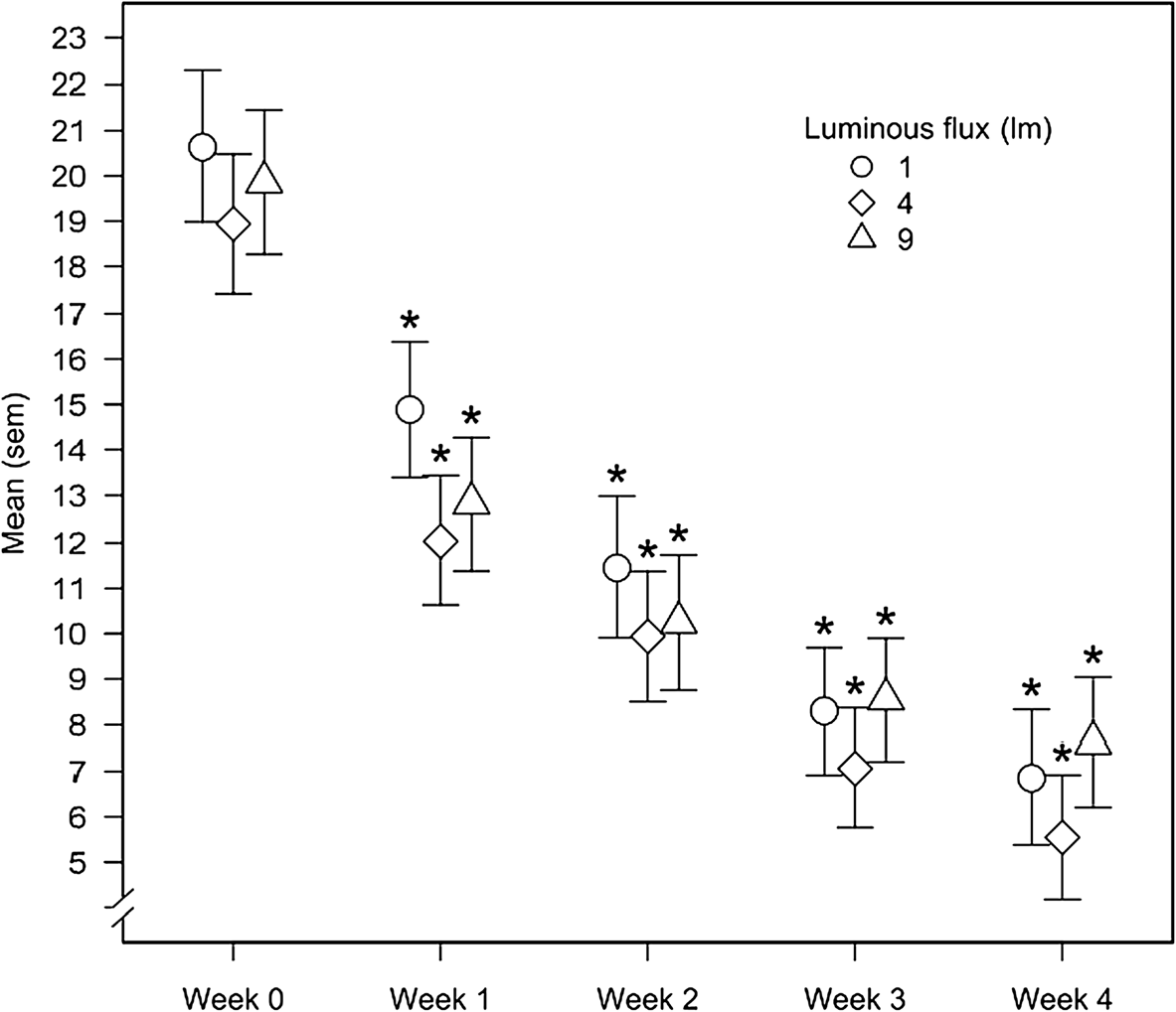
**BDI sum scores during the four-week treatment period.** Values are reported as the mean ± sem.

The self-rated BDI was assessed weekly to evaluate patients’ depressive symptoms throughout the study (Figure 4). A statistically significant decrease was found in each treatment group after one week (time point week 1) when compared with baseline, and the decrease in symptoms continued throughout the study. There was no significant difference between the reductions in depression scores between treatment groups.

### Cognitive performance

The cognitive performance of the patients, as measured using the TMT-A, improved significantly in all three treatment groups. On the TMT-B test, a significant improvement in total time was observed in group 2 only. Total performance times and absolute and percent improvements are presented in Table 5 for all three treatment groups.

**Table 5:** **The time in seconds required to complete the Trail-making test (TMT) part A and B**

**Measure**	**Group 1 (n = 28)**			**Group 2 (n = 31)**			**Group 3 (n = 30)**		
	**Mean (s)**	**95% CI (s)**		**Mean (s)**	**95% CI (s)**		**Mean (s)**	**95% CI (s)**	
TMT-A									
Week 0	31.0	27.0-34.9		34.5	31.3-37.6		32.7	26.1-39.2	
Week 4	27.0	23.3-30.7		27.1	24.5-29.8		22.5	19.5-25.4	
TMT-B									
Week 0	65.1	57.4-72.7		66.8	60.7-72.8		73.0	62.0-84.1	
Week 4	59.7	49.4-70.0		56.6	51.8-61.4		58.5	47.5-69.5	
Change	**%**	**95% CI**	**p-value**	**%**	**95% CI**	**p-value**	**%**	**95% CI**	**p-value**
TMT-A	−9.7	−2.2- -17.3	0.0066	−18.0	−11.6- -24.4	<0.0001	−18.7	−12.3- -25.1	0.0004
TMT-B	−6.5	5.0- -18.1	0.1104	−11.2	−4.9- -17.4	0.0009	−7.9	−1.2- -16.9	0.1060

### Adverse events

The percentage of patients who reported potential bright light-related adverse events was 28.1% (n = 25). There were no statistically significant differences in the presence of bright light-related adverse events between treatment groups. The most common adverse events reported were headache, insomnia, and nausea, which were reported by 10.1%, 5.6%, and 3.4% of the patients, respectively. In addition, patients reported dizziness, earache, abnormal sensation in the maxillary region, tinnitus, tiredness, irregular heartbeat, and irritability. The total number of different adverse events is presented in Table 6.

**Table 6:** **The total amount of treatment-related adverse events**

**Adverse event**	**Total emergence**	**Amount in group1**	**Amount in group2**	**Amount in group3**	**p**
Headache	9	4	3	2	ns
Insomnia	5	2	1	2	ns
Nausea	3	2	0	1	ns
Dizziness	2	0	1	1	ns
Tinnitus	2	1	0	1	ns
Tiredness	2	1	1	0	ns
Earache	3	0	1	2	ns
Irritation	1	0	1	0	ns
Abnormal sensation in the maxillary region	1	0	0	1	ns
Irregular heart beat	1	1	0	0	ns
**Total**	**29**	**11**	**8**	**10**	ns

## Discussion

In the present study, both the self-rated and psychiatrist-rated depressive and anxiety symptoms of SAD patients decreased significantly during the four-week study period, even after adjusting for age and gender. However, there were no significant differences in the improvement of depressive or anxiety symptoms between groups receiving different intensities of bright light via the ear canals.

The mean decrease in BDI scores was 12.9 points (66%), from a moderate depression level to a minimal depression level [50], in the three treatment groups. The psychiatrist-rated SIGH-SAD and HAMA scores decreased 16.8 points (46%) and 11.2 (49%), respectively. HAMA scores decreased from a moderate anxiety level to a normal level [51] during the four-week study period in each treatment group.

Approximately three out of four (76%) patients in this study fulfilled the BDI response criterion, which was defined as a decrease of symptoms of at least 50%. The mean SIGH-SAD and HAMA response rates were 39% and 52% in the present study. Remission rate was defined as a total score ≤ cut-off and a decrease of symptoms by 50% or more. The mean BDI remission rate was 75% for our study, whereas the SIGH-SAD remission rate varied from 13% to 29% in the three treatment group.

The alleviation of symptoms was somewhat lower in the present study compared with our previous study [42]. This result was due possibly to a greater placebo response in the earlier study resulting from daily treatment visits at the research center and the related person-to-person interactions, which are known to play a role in the placebo effect. Nonetheless, the significant reduction in depressive and anxiety symptoms observed in the present study parallels earlier studies that used conventional bright light therapy devices and pharmacological therapy in the treatment of SAD and anxiety [13,14,21,56-62].

In earlier studies on patients suffering from SAD, symptoms usually have been measured by inventories, which concentrate on measurements of depression domains. Because anxiety commonly co-occurs and has a neurochemical similarity to depression [63], the measurement of anxiety symptoms is justified in the present study. The use of an anxiety measurement in this study is a significant strength over earlier studies that have investigated treatments for SAD.

The remission rates measured by the SIGH-SAD are also slightly lower in the present study compared with earlier bright light studies that used conventional bright light treatment [13,21,59]. It is known that SAD patients, who experience more severe symptoms, have lower remission rates [64,65]. The mean total baseline score in the present study was 36. Based on the assessment at baseline, the SIGH-SAD score would have had to decrease by at least 28 points (78%) in order to fulfil our previously defined criterion for remission (≤8). Thus, the high baseline score may be partially responsible for the low number of patients who met the criterion of remission for the SIGH-SAD in this study. In addition, contrary to most of the earlier SAD studies, patients suffering from bipolar disorder were excluded from this study, which may have reduced the occurrence of spontaneous remissions.

Cognitive deficits are amongst the symptoms reported for depression [9], and neuropsychological deficits in depressed patients may be present themselves as impaired performance, e.g., in attention and executive function [66]. Earlier studies with pharmaceuticals [67] or conventional bright light therapy [9] have reported deficits in cognitive performance even after the remission of depressive symptoms. In this study, cognitive performance was found to improve significantly in all three treatment groups measured by TMT-A, and for the medium dosage group for TMT-B. At the end of the four-week trial, the time required to complete the tests were comparable to the values of healthy individuals [53] in all groups. The earlier findings on effect of TBL on cognitive performance in healthy subjects [40,41] were also observed after treatment period of several weeks. In a recent study TBL was not found to have an acute effect on reaction time [45] suggesting that the improved cognitive performance found in this study is instead due to cumulative effect or associated with an improvement in depressive symptomatology.

The most common side effects observed in this study were headache, insomnia, and nausea at a rate of 28%, which is comparable to the rate reported in earlier bright light studies using conventional bright light devices (e.g., 25% [68]). In earlier studies that used conventional BLT, the presence of headaches was slightly lower, whereas insomnia and nausea were reported more often [68]. However, it is important to note that none of the patients in the study stopped their participation due to the presence of adverse effects.

One strength of our study is that we used both observe rating SIGH-SAD and self-rating BDI to assess the severity of depressive symptoms. SIGH-SAD as a sum of HAM-D and atypical scores covers symptoms of both atypical and melancholic depression, while atypical symptoms are far less relevant in the BDI version used in this study [69]. HAM-D and BDI overlap on only half of the items. These scales therefore rate different aspects of the disease and thus poor concordance between scores might be predicted [70]. Patient or observer biases, severity of illness, age, education, sex, and personality variables affect the level of correlation between observer and self-rating scales [70–72]. These aforementioned issues may cause differentiated scores on the SIGH-SAD and BDI inventories. Thus, comparisons between scales should be conducted with caution [70].

One limiting factor of our study is the lack of a proper control group. The lack of a true placebo condition for visible light interventions is the greatest challenge for studying the effects of bright light therapy [57,73,74]. The bright light used in this study was visible to the patients even though it was administered using extra-visual routes via the ear canals. Because the treatment was conducted at home without supervision by the investigators, it was not possible to create a proper placebo condition in this study.

It is well known that bright light treatment is accompanied by a placebo response [14,75]. In meta-analysis on earlier antidepressant studies the size of the placebo effect was found to be 29.7% [76] and account for 68% of the effect in the treatment group [77]. It is assumed that sham devices have higher response rates than placebo pills in the treatment of depression [78]. However, repetitive transcranial magnetic stimulation (rTMS) as a non-pharmacological and transcranially administered treatment has been found to elicit even lower placebo response than pharmacological therapy in major depression [79]. In addition the subjects in this study were severe depressed, which decreases the effect of placebo [80]. Assuming that the placebo response of transcranial bright light is in line with the placebo effect found in earlier antidepressant studies, it is not likely that the alleviation of the symptoms in this study would be entirely explained by means of placebo effect. In future studies, the size of the placebo response should be carefully scrutinized. In addition, inclusion of the follow-up assessments after the end of the treatment would deliver valuable information about the sustainability of the induced effects on mood and cognition.

The improvements in anxiety and depressive symptoms for the lowest dosage group paralleled the improvements found in the other two dosage groups. Hence, a dose-response relationship between the different light intensities was not observed. The earlier findings concerning intensity-based dose-response relationships of phototherapy are contradictory. An intensity-based dose-response relationship has been reported in earlier studies that used conventional fluorescent bright light sources [81]. However, bright white light intensity of 250 lux has been found to be as effective as 10000 lux when the light is administered early in the morning [13,21]. In addition, the spectral characteristics of light have been shown to influence the effect of light treatment in SAD [19,82]. Blue-enriched white-light (750 lux) and white light (10000 lux) have been proven to be equally effective in alleviating SAD symptoms [57]. Blue-enriched light of even lower intensity (98 lux) has also been shown to be effective in the treatment of SAD [19]. Furthermore, a recent study has shown that narrow-bandwidth blue light is effective in the treatment of SAD [82]. Because illuminance measurements in lux or lumens assume a photopic light sensitivity peaking at 555 nm, the illuminance values given in lux or lumen units underestimate the actual illuminance of blue-enriched light sources. Based on the literature on the wavelength-dependent effects of light in humans, a blue-enriched light source was used in this study. It is not unreasonable to assume that the recently found extra-retinal proteins such as melanopsin [83] have similar features as the ones located in the retina and thus are also sensitive to shorter wavelengths.

The light intensity at which the SAD light-therapy response reaches saturation is still under discussion [57]. Based on the results from current studies that have used blue-enriched light, the effect of light saturates at fairly low light intensities [57]. The lack of a dose response in therapeutic changes in the current study may therefore be a consequence of the light saturation. This notion is supported by previous non-clinical bright light dose-response studies that have shown that half of the maximum alerting effect and phase resetting effect of a light pulse of 9100 lux can be achieved with 100 lux [84,85]. The data of Chang et al. [86] have also shown that per minute of exposure a 0.2-hr light pulse of 10000 lux was five times more efficient in resetting the circadian pacemaker than a 4-hr light pulse of the same intensity [86]. Moreover, a recent study failed to find a dose response between 10000 lux and 2000 lux bright light treatment on depression and anxiety symptoms [87].

Another limiting factor of our study is that the dose response was examined only as an intensity-dependent variable, while the duration of treatment is also known to have an effect on the response [88]. This latter effect needs to be examined in a duration-based dose response study. In addition, the exact timing of the treatment and a detailed analysis of the sleep/wake cycles of patients was not included in the current study. The only instructions that the patients received regarding the timing of light treatment was to administer the treatment shortly after habitual wake time and before noon each day.

To date, there are no studies that describe the penetration of BLT to areas of the human brain, although there is evidence that light is able to penetrate the skull [37,38]. The distance from the light source to the nearest brain area is approximately 2 cm. The brain regions which might encounter most of the light from the ear canal are the anterior cerebellum, the inferior temporal lobe, and the pons in the brainstem. The midbrain in the brainstem, the posterior diencephalon, and the anterior occipital lobe may also be within the range of the light photons, especially at longer wavelengths [39]. Haemoglobin and skin melanin are the main absorbers of visible light [89], while bone structures and many types of cells primarily scatter light [90]. The relative absence of skin melanocytes and the small amount of blood haemoglobin in the ear keeps the light absorption in this design at a minimum before entering brain tissue [39].

There is emerging evidence that at least some of the non-image forming effects of bright light may be mediated transcranially without affecting the eyes. However, there are a limited number of studies that investigate this issue. Furthermore, the findings of these studies are preliminary and not all of them have been conducted in a placebo setting. In addition, the putative role of the brain opsins in phototransduction is still unknown. Thus, the findings of this study should be interpreted cautiously and further studies on the effects of transcranial bright light with an adequate placebo condition are needed.

## Conclusions

In sum, the results of this study suggest that transcranial bright light treatment may have antidepressant and anxiolytic effects on patients suffering from SAD. The present results are comparable with the findings of previous bright light studies that have used conventional bright light devices or pharmaceuticals in the treatment of depressive and anxiety symptoms. In future, studies on the efficacy and mechanisms of action of transcranially administered bright light are needed. Although the effects of transcranial light are not likely to be mediated via the eyes, the effects of TBL should also be studied in totally blind people. Further studies are also needed to measure the duration-based dose response.

## Abbreviations

BDI: Beck depression inventory
BLT: Bright light therapy
DSM: Diagnostic and statistical manual of mental disorders
HAMA: Hamilton anxiety rating scale
HAMD: Hamilton depression rating scale
MINI: Mini International Neuropsychiatric Interview
LED: Light-emitting diode
ipRGC: Intrinsically photosensitive retinal ganglion cell
NIF: Non-image forming
OPN3: Encephalopsin
OPN4: Melanopsin
OPN5: Neuropsin
RHT: Retinohypothalamic Tract
SAD: Seasonal affective disorder
SCN: Suprachiasmatic nucleus
SIGH-SAD: Structured interview guide for the Hamilton depression rating scale – seasonal affective disorder
TBL: Transcranial bright light
TMT: Trail making test
